# Aldo-keto reductase family 1 member A1 (AKR1A1) exerts a protective function in alcohol-associated liver disease by reducing 4-HNE accumulation and p53 activation

**DOI:** 10.1186/s13578-024-01200-0

**Published:** 2024-02-03

**Authors:** Ying-Wei Lan, Wan-Ru Chen, Gary Ro-Lin Chang, Ying-Cheng Chen, Kowit-Yu Chong, Kai-Cheng Chuang, Yung-Tsung Kao, Ming-Shan Chen, Chuan-Mu Chen

**Affiliations:** 1 Department of Life Sciences, and Doctoral Program in Translational Medicine, College of Life Sciences, National Chung Hsing University, Kuo Kuang Rd, Taichung, 402 Taiwan; 2 Division of Pulmonary Biology, Cincinnati Children’s Hospital Medical Center, University of Cincinnati, Cincinnati, OH USA; 3 Department of Medical Biotechnology and Laboratory Science, College of Medicine, Chang Gung University, Taoyuan, 333 Taiwan; 4 Hyperbaric Oxygen Medical Research Lab, Bone and Joint Research Center, Chang Gung Memorial Hospital, Taoyuan, 333 Taiwan; 5 The iEGG and Animal Biotechnology Center, National Chung Hsing University, Taichung, 402 Taiwan; 6 Department of Anesthesiology, Ditmanson Medical Foundation Chia-Yi Christian Hospital, Chia-Yi, 600 Taiwan; 7 Rong Hsing Research Center for Translational Medicine, National Chung Hsing University, Taichung, 402 Taiwan

**Keywords:** Alcohol-associated liver disease (ALD), Aldo-keto reductase family 1 member A1 (AKR1A1), 4-HNE, p53, Lipid accumulation, Liver fibrosis

## Abstract

**Background:**

The development of alcohol-associated liver disease (ALD) is influenced by the amount and duration of alcohol consumption. The resulting liver damage can range from reversible stages, such as steatosis, steatohepatitis and alcoholic fibrosis, to the advanced and irreversible stage of cirrhosis. Aldo-keto reductase family 1 member A1 (AKR1A1) is a member of the aldo-keto reductase family that catalyzes the reduction of aldehyde groups to their corresponding alcohols in an NADPH-dependent manner. AKR1A1 was found to be downregulated in patients diagnosed with ALD. This study aims to interpret the protective effects of AKR1A1 on the development of ALD.

**Methods:**

A 5% alcohol-fed (AF) *Akr1a1* knockout (*Akr1a1*^−/−^) mouse model and an AML12 hepatocyte model were used. The effects of AKR1A1 on liver function, inflammation, oxidative stress, lipid accumulation, and fibrosis were assessed by ELISA, western blotting, RT‒PCR, and a variety of histological staining methods in AF-induced wild-type (WT) and *Akr1a1*^*−/−*^ mice compared to control liquid diet-fed (PF) WT and *Akr1a1*^*−/−*^ mice.

**Results:**

The results demonstrated that AF-WT mice expressed higher levels of AKR1A1 than WT mice fed a control diet, and they did not show any noticeable liver steatosis. However, AF-*Akr1a1*^−/−^ mice displayed a lower survival rate and more severe liver injury than AF-WT mice, as demonstrated by increased proinflammatory cytokines, oxidative stress, lipid accumulation, fibrosis, and reduced antioxidant enzymes in their livers. Additionally, elevated levels of 4-HNE and p53 phosphorylation were observed in AF-*Akr1a1*^−/−^ mice, suggesting that the loss of AKR1A1 led to increased 4-HNE accumulation and subsequent activation of p53, which contributed to the progression of ALD. Furthermore, in AML12 hepatocytes, *Akr1a1* knockdown aggravated oxidative stress and steatosis induced by palmitic acid/oleic acid (P/O) inflammation induced by lipopolysaccharide (LPS), and fibrosis induced by TGF-β1.

**Conclusions:**

This loss-of-function study suggests that AKR1A1 plays a liver-protective role during chronic alcohol consumption by reducing the accumulation of 4-HNE and inhibiting 4-HNE-mediated p53 activation.

**Supplementary Information:**

The online version contains supplementary material available at 10.1186/s13578-024-01200-0.

## Background

Alcohol-associated liver disease (ALD) is considered to be one of the leading causes of liver disease-related mortality. It accounts for approximately two million deaths annually worldwide, and this number is continuously rising [1]. The clinical characteristics of ALD cover a broad spectrum of liver lesions ranging from reversible fatty liver, steatohepatitis, and fibrosis to irreversible cirrhosis, primarily due to chronic liver inflammation caused by excessive consumption of alcohol. More than 65% of ALD patients diagnosed with cirrhosis die within 5 years [2]. Activation of the immune system, increased gut-derived endotoxins, imbalances in pro-oxidant production and antioxidant defenses, hepatocyte apoptosis and necrosis, and fibrosis are now regarded as the primary factors in the pathogenesis of ALD [3–5]. Although the pathogenesis of ALD has been extensively studied, the related gene markers and their underlying molecular mechanisms that influence several signaling pathways, such as oxidative stress, inflammatory responses, hormone regulation, and hepatocyte death remain limited. Therefore, more in-depth research in this context is required for the discovery of a novel interventional therapy or strategy to halt the progress of ALD.

Several enzymes have been found to be involved in ethanol metabolism in the mammalian liver. Alcohol dehydrogenase (ADH) and cytochrome P450 2E1 (CYP2E1) are alcohol-oxidizing enzymes that are expressed in the cytosol and microsomes of hepatocytes, respectively, and catalyze the conversion of ethanol into acetaldehyde, which results in damage caused by ethanol consumption. Moreover, acetaldehyde dehydrogenase (ALDH) further oxidizes acetaldehyde to acetate in an NAD^+^-dependent manner in the mitochondria of hepatocytes [6]. In fact, endogenous aldehydes can be produced during the metabolism of lipids, carbohydrates, amino acids, vitamins, and steroids. These endogenous aldehydes are highly reactive electrophiles that interact with DNA, proteins, and phospholipids leading to a variety of cytotoxic, mutagenic, or carcinogenic consequences. Therefore, the detoxification of aldehydes is essential in various normal cells and tissues. Aldo-keto reductase family 1 member A1 (AKR1A1), also known as aldehyde reductase, is a member of the AKR superfamily that is constitutively expressed in the liver, where it acts as a detoxifying enzyme to catalyze the NADPH-dependent biotransformation of a range of aromatic and aliphatic aldehydes to corresponding metabolites [7].

Previous studies demonstrated that AKR1A1 plays roles in drug metabolism (e.g., doxorubicin, tiaprofenic acid) and prostaglandin F2 biosynthesis and acts as a suppressor of diabetic complications. However, it was observed to be overexpressed in many cancer cells and plays antithetical roles in cancer development [8]. A novel function of AKR1A1 has been identified, it regulates protein S-nitrosylation from yeast to mammals [9]. This function was further demonstrated to exert a protective effect against acute kidney injuries [10]. In addition, AKR1A1 catalyzes the conversion of glucuronate (GlucA) to gulonate, which is a key step in the synthesis of L-ascorbic acid (AsA or vitamin C) in mice [11], whereas the pathway of AsA synthesis is defective in primates. Recently, the AKR1A1 735G > A variant was found to cause GlucA accumulation in patients with schizophrenia, a psychiatric disorder [12]. In *Akr1a* knockout mice (*Akr1a*^*−/−*^) studies, AKR1A1 was reported to exert a protective effect against carbon tetrachloride (CCl_4_)-induced [13], acetaminophen-induced [14–16], and N-nitrosodiethylamine-induced [17] hepatic injuries by replenishing AsA via its antioxidative activities. However, *Akr1a*^*−/−*^ mice were shown to be resistant to thioacetamide (TAA)-induced liver damage by suppressing the endoplasmic reticulum (ER) stress-induced apoptosis pathway, with no relevance to AsA levels in plasma [18]. These studies show that the functions and mechanisms mediated by AKR1A1 may vary among diverse cell types or tissues. Currently, the clinical role and relevance of AKR1A1 in ALD remain unclear.

The present study aimed to investigate whether endogenous AKR1A1 levels affect the progression of ALD and unravel the underlying mechanism through the use of an in vivo chronic alcohol feeding mouse model and an in vitro AML12 hepatocyte model.

## Materials and methods

### Animals and chronic alcohol feeding model

**Fig. 1:**
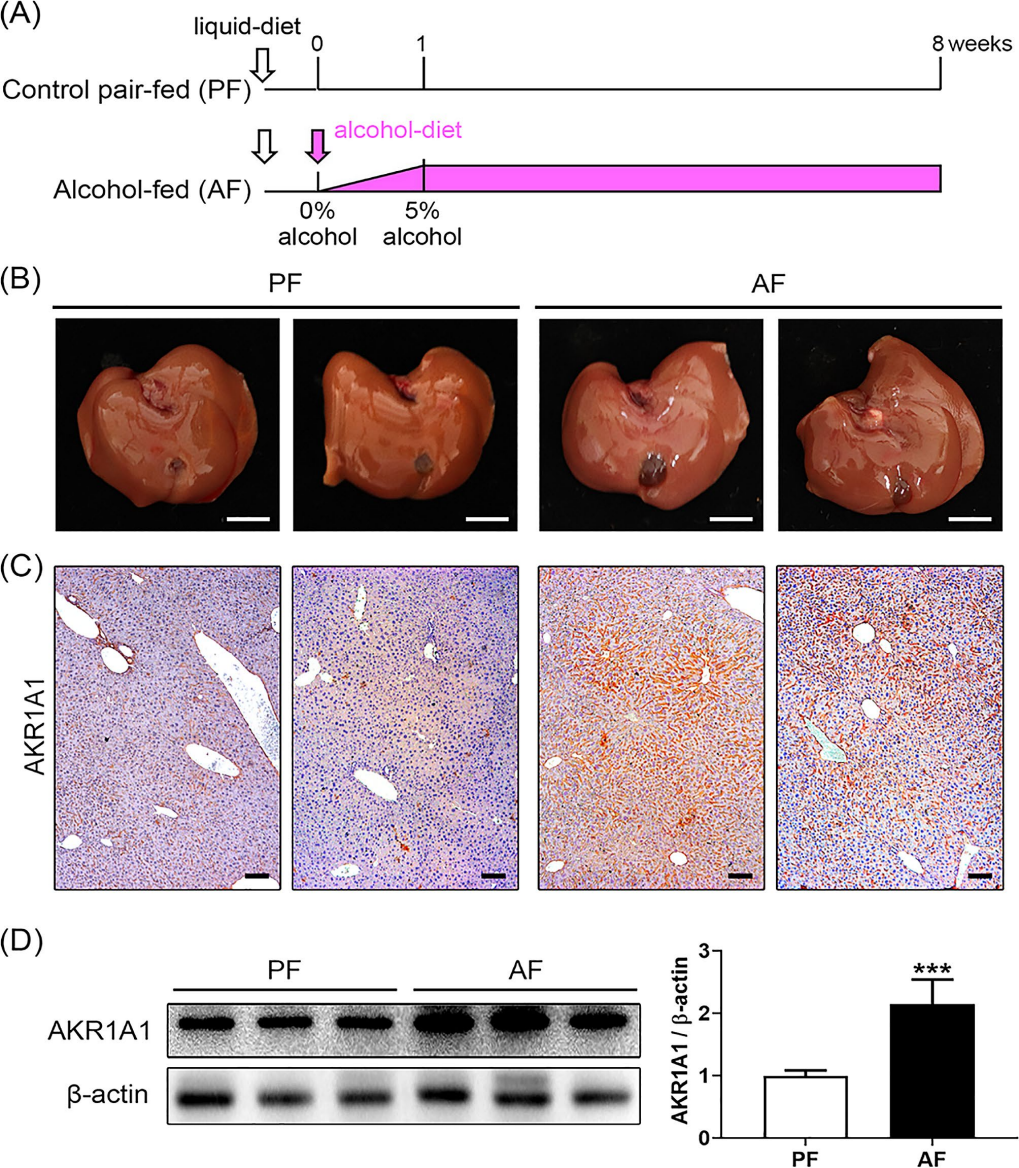
The present chronic alcohol feeding model is depicted in Panel (**A**). The experiment was conducted in both male wild-type (WT) ICR and *Akr1a1*^*−/−*^ mice; these mice were orally administrated two different treatments: the control pair-fed (PF) and alcohol-fed (AF) treatments. The alcohol feeding regimen was performed by a stepwise increase in the alcohol content to 5% in the first week and then maintained at that dose for seven weeks. After the treatment, the mouse livers were removed for analysis. The gross liver appearances of WT mice are shown in Panel (**B**) (scale = 1 cm). The liver tissue sections subjected to IHC staining for the AKR1A1 protein are shown in Panel (**C**) (scale bars = 100 μm). The liver protein lysates subjected to western blot analysis for the detection of AKR1A1 levels are shown in Panel (**D**), in which the quantification (right panel) was performed by β-actin normalization. Data are expressed as the means ± SD (*n* = 6), ***p < 0.001

The present animal experiment used male AKR1A1 knockout (*Akr1a1*^*−/−*^, ICR background) mice and wild-type (WT) ICR mice. The *Akr1a1*^*−/−*^ mouse line was previously established in our laboratory by pronuclear microinjection [19], and ICR mice were purchased from BioLASCO Co., Ltd., Taipei, Taiwan. The procedures for chronic alcohol consumption used a long-term Lieber-DeCarli diet without binges as previously described [20]. At the age of eight weeks, *Akr1a1*^*−/−*^ and WT ICR mice were given either Lieber-DeCarli liquid diets containing ethanol (alcohol-fed; AF) (*Akr1a1*^−/−^ + AF, *N* = 12; WT + AF, *N* = 6) or an isocaloric maltodextrin control liquid diet (pair-fed; PF) (*Akr1a1*^−/−^ + PF, *N* = 6; WT + PF, *N* = 6) for 8 weeks. The feeding was carried out through the use of glass liquid diet feeding tubes. The ethanol content in the liquid diet was gradually increased at an interval of two days from 1%, 2%, and 4% to a final of 5% in the first week, and then this percentage was maintained for 7 weeks (Fig. 1A). Diets were refreshed every day, and the volumes consumed were recorded. At the end of 8 weeks of feeding, mice were anesthetized using isoflurane, and blood was collected by cardiac puncture for the preparation of serum samples. Then, the epididymal fat and the whole livers were removed and stored at -80 °C for subsequent analyses.

### Biochemical analysis

The serum levels of aspartate aminotransferase (AST) and alanine aminotransferase (ALT) were measured using an IDEXX VetTest Chemistry Analyzer (IDEXX Laboratories Inc., Westbrook, ME, USA). The triglyceride content was measured in 100 mg of liver tissue using a triglyceride quantification colorimetric kit from BioVision (Milpitas, CA, USA). The contents of glutathione (GSH) and oxidized glutathione (GSSG) and the content of malondialdehyde (MDA) were measured in 100 mg of the liver tissues using a GSH assay kit and a TBARS assay kit from Cayman Chemical (Ann Arbor, MI, USA), respectively. All of the measurements were conducted in accordance with the manufacturer’s instructions.

### Quantitative reverse transcription-polymerase chain reaction (qRT–PCR)

The extraction of total RNA from liver tissues and cell cultures was performed using a Presto™ DNA/RNA/Protein Extraction Kit (Geneaid, Taipei, Taiwan). Subsequently, 1‒5 µg of total RNA was reverse transcribed into single-stranded cDNA using an MMLV Reverse Transcription kit (Protech, Taipei, Taiwan). The following qPCR was performed by mixing single-stranded cDNA, target gene primers (Supplementary Table S1), and 2X qPCR SyGreen Mix (Protech) in a total volume of 20 µl in a 384-well PCR plate and then analyzed using a QuantStudio 6 Pro Real-Time PCR System (Applied Biosystems, Waltham, MA, USA). The PCR program was run at 95 ^o^C for 2 min, with 50 cycles at 95 ^o^C for 15 s, 65 ^o^C for 15 s, and 72 ^o^C for 10 s accompanying signal acquisition, followed by a melting curve detection from 65 ^o^C to 95 ^o^C. The relative mRNA levels were calculated by the 2^−∆∆Cq^ method with β-actin normalization, where Cq represents the threshold cycle number.

### Western blot analysis

The total protein concentration of each sample was determined by the BCA method prior to western blot analysis. Afterward, 50 µg of protein was loaded and separated by SDS-polyacrylamide gel electrophoresis (SDS**–**PAGE). Then, the proteins were then transferred to a polyvinylidene difluoride (PVDF) membrane. The membrane was blocked with 5% skim milk in Tris-buffered saline with 0.1% Tween 20 (TBST) for 1 h at room temperature and probed overnight at 4 ^o^C with the primary antibodies (Supplementary Table S2). The next day, the membrane was subjected to three 10-min TBST washes. After incubation with the diluted HRP-conjugated secondary antibodies for 2 h, it underwent another three 10-min TBST washes. Finally, the membrane was rinsed with the substrate reagent and subjected to detection using an ImageQuant LAS 4000 mini luminescent image analyzer (GE Healthcare, Chicago, IL, USA). The intensities of target proteins were quantified using ImageJ software, and the relative protein levels were calculated by β-actin normalization.

### Histological assessments

For histological analysis, one lobe of liver tissue was fixed with 10% formalin overnight and embedded in paraffin. The embedded tissue block was sectioned at a thickness of 5-µm and stained according to standard laboratory protocols for hematoxylin-eosin (H&E), Masson’s trichrome, and Sirius red staining, as previously described [21]. Steatosis was evaluated by oil red O (ORO) staining of 10-µm sections from snap-frozen OCT-embedded liver tissue and 10% formalin-fixed cells according to a standard protocol as previously described [22]. For immunohistochemical staining (IHC), the paraffin-embedded liver sections were dewaxed, rehydrated, and incubated with a 200-fold dilution of anti-4-HNE and anti-AKR1A1 primary antibodies overnight at 4 °C. Then they were subjected to further staining with a Novolink™ Max Polymer Detection System (Leica Biosystems, Wetzlar, Hesse, Germany). Representative images were captured by using an Olympus IX71 microscope with an AxioCam MRc camera. The positive areas for Masson’s trichrome, Sirius red, and ORO staining were quantified using ImageJ software with the plug-in IHC toolbox (National Institutes of Health, Bethesda, MD, USA).

### AML12 cell culture, AKR1A1 knockdown, and in vitro cellular models

The AML12 cell line, which are hepatocytes isolated from the normal liver of a mouse, was purchased from the Bioresource Collection and Research Center, Hsinchu, Taiwan. AML12 cells were maintained in DMEM/F-12 medium supplemented with 10% fetal bovine serum (FBS), 10 µg/ml of insulin, 5.5 µg/ml of transferrin, 5 ng/ml of selenium, 40 ng/ml dexamethasone, and 1% penicillin/streptomycin solution [23].

The lentiviral small hairpin RNA (shRNA) clones TNCN0000042319 (targeting the mouse *Akr1a4* gene) and TRCN0000006180 (targeting the human *PGK1* gene) were purchased from the RNA Technology Platform and Gene Manipulation Core, Taipei, Taiwan (https://rnai.genmed.sinica.edu.tw). The first shRNA clone, designated sh-AKR1A1 in this work, was used to knock down the expression of AKR1A (aldehyde reductase) in AML12 cells, while the second shRNA clone, designated sh-control, served as a control for the RNA interference (RNAi) experiments. RNAi-related experiments were performed by infecting AML12 cells with lentiviral shRNA clones at a multiplicity of infection (MOI) of 10 in the presence of polybrene (8 µg/ml) for 24 h, followed by puromycin (8 µg/ml) selection. The shRNA-treated AML12 cultures were subjected to the following experiments.

To generate an in vitro model of alcoholic-induced liver steatosis, 1 mM palmitic acid and oleic acid mixture (P/O, 2:1) was applied to shRNA-treated AML12 cells for 72 h. After the treatment, cells were either collected for qRT**–**PCR analysis or subjected to 10% formalin fixation and ORO staining. To generate in vitro models of liver inflammation and fibrosis, 1 µg/ml LPS and 5 ng/ml TGF-β1 were applied to shRNA-treated AML12 cells for 24 and 48 h, respectively. Subsequently, cells were collected for qRT**–**PCR analysis to examine the related gene expression in AML12 cells.

### Statistical analysis

The data are presented as the mean ± SD and were plotted using GraphPad Prism 8 software (San Diego, CA, USA). The statistical analysis between two groups was performed using a two-tailed Student’s *t* test, while one-way ANOVA with Tukey’s post hoc test was used for multiple-group comparisons. A *p* value < 0.05 was considered to indicate statistically significant differences.

## Results

### Downregulation of AKR1A1 in clinical ALD patients

First, we investigated the expression patterns of the *Akr1al* gene and ALD from previous transcriptomic and proteomic studies. At the transcriptional level, several members of the AKR1 family were found to be significantly differentially expressed in alcoholic hepatitis patients; the *Akr1al* gene was downregulated by 0.804-fold compared to normal individuals (Supplementary Table S3) [24]. The epigenetic data showed that hypermethylation was found at the promoter, 5’-UTR, and exon regions of the *Akr1al* gene in livers with alcoholism-induced cirrhosis compared to normal livers (Supplementary Fig. S1) [25]. At the protein level, data mining from a liver proteome of an ALD cohort study identified significantly reduced AKR1A1 levels in liver tissues at the advanced fibrosis stages of F3 and F4 (Supplementary Table S4) [26]. Overall, the downregulation of the *Akr1a1* gene in the above clinical studies prompted us to further investigate the key role of AKR1A1 in the progression of ALD and the underlying mechanisms.

### An increase in AKR1A1 levels reduces the risk of developing alcohol-induced liver damages

To explore the protective role of AKR1A1 during chronic alcohol consumption, a chronic alcohol-feeding mouse model was created by feeding *Akr1a1*^*−/−*^ and WT mice a 5% alcohol-containing (AF) and a control liquid diet (PF) for 8 weeks (Fig. 1A). Thereafter, physiological data, the expression of AKR1A1, and the related changes in livers were compared. At the end of treatment, no significant differences in body and liver weight changes were found in either PF- or AF-treated WT mice (Supplementary Table S5), and the liver exhibited a normal appearance in both groups (Fig. 1B). Nevertheless, a significant reduction in epididymal fat was recorded in the AF-treated mice, which was consistent with previous animal studies showing that chronic alcohol feeding stimulates hyperlipolysis in white adipose tissues and increases hepatic fat accumulation in ALD [27, 28].

We next investigated whether there was a difference in hepatic AKR1A1 levels between PF- and AF-treated WT mice (Fig. 1C, D). A striking increase in AKR1A1 levels was detected in the livers of the AF-treated WT mice compared to the PF-treated WT mice by immunohistochemical staining (Fig. 1C), and significant increases in AKR1A1 intensities were detected by western blot analysis (Fig. 1D), implying that WT mice avoid alcohol-associated liver damage by means of increasing AKR1A1 levels.

### Loss of AKR1A1 function exacerbates alcohol-induced liver injury and inflammation

**Fig. 2:**
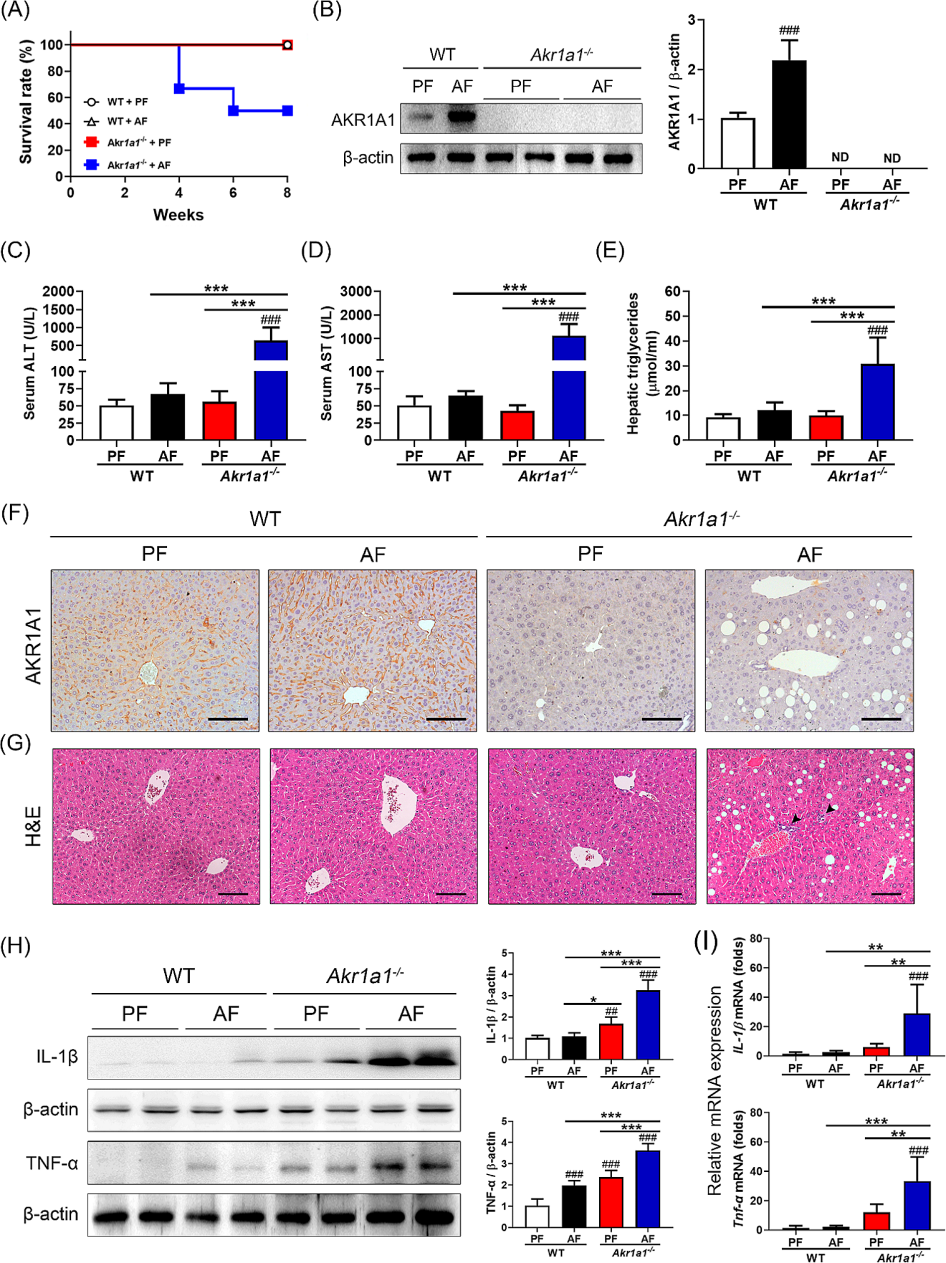
Loss of AKR1A1 exacerbates alcohol-induced liver injury and inflammation. After the treatments, the survival rates (**A**) were recorded, and western blot analyses of hepatic AKR1A1 expression (**B**), serum assays of ALT (**C**) and AST (**D**) levels, hepatic triglyceride content assays (**E**), IHC staining for hepatic AKR1A1 expression (**F**), H&E staining of the liver tissue sections (**G**), western blot analyses of hepatic IL-1β and TNF-α levels (**H**), and qRT‒PCR examination of hepatic IL-1β and TNF-α mRNA levels (**I**) were performed. The data were compared between WT and *Akr1a1*^*−/−*^ mice receiving the PF and AF treatments. The quantification of the western blot and qRT‒PCR was performed by β-actin normalization, and the relative protein and mRNA expression were compared to the PF-treated WT mice in folds. Data are represented as the means ± SD (*n* = 6). Statistical differences were marked as follows: ^#^*p* < 0.05, ^##^*p* < 0.01, ^###^*p* < 0.001 (compared with the PF-treated WT group); *p < 0.05, **p < 0.01, ***p < 0.001 (compared between the other groups)

In contrast, the mortality rate of the AF-treated *Akr1a1*^*−/−*^ mice was 50%, and the survival rate of the PF-treated *Akr1a1*^*−/−*^ mice and both PF- and AF-treated WT mice at the end of the study was 100% (Fig. 2A). Moreover, AF-treated *Akr1a1*^*−/−*^ mice showed a significant loss in body weight (-1.42 g on average) at the end of alcohol feeding compared to their initial weight; however, in the other groups, body weights were significantly increased (WT + PF: +3.66 g; WT + AF: +2.19 g; *Akr1a1*^*−/−*^*+*PF: +3.66 g on average). In addition, a notable increase in liver weight and the liver-to-body weight ratio was recorded in AF-treated *Akr1a1*^*−/−*^ mice, accompanied by a striking decrease in their epididymal fat weight and epididymal fat-to-body weight ratio (Supplementary Table S5). No observable AKR1A1 signals were identified in the livers of AF- and PF-treated *Akr1a1*^*−/−*^ mice (Fig. 2B, F). Serum ALT and AST levels were approximately 10 and 20 times higher in AF-treated *Akr1a1*^*−/−*^ mice than in the other groups (Fig. 2C, D), demonstrating liver dysfunction in those mice. Hepatic triglyceride levels were increased 3-fold in the AF-treated *Akr1a1*^*−/−*^ group compared to the other groups, suggesting excessive fat deposition in the livers (Fig. 2E). The IHC and H&E images showed increased fat droplets and inflammatory cell aggregates in the liver sections of AF-treated *Akr1a1*^*−/−*^ mice (Fig. 2F, G). The expression of TNF-α and IL-1β, major proinflammatory cytokines, was significantly increased at both the transcriptional and protein levels in the livers of AF-treated *Akr1a1*^*−/−*^ mice compared to those of PF-treated *Akr1a1*^*−/−*^ mice and both PF- and AF-treated WT mice (Fig. 2H, I). These data demonstrated that the loss of AKR1A1 function deteriorates liver injury and inflammation in AF-treated *Akr1a1*^*−/−*^ mice.

### Loss of AKR1A1 function increases alcohol-induced oxidative stress in the liver

**Fig. 3:**
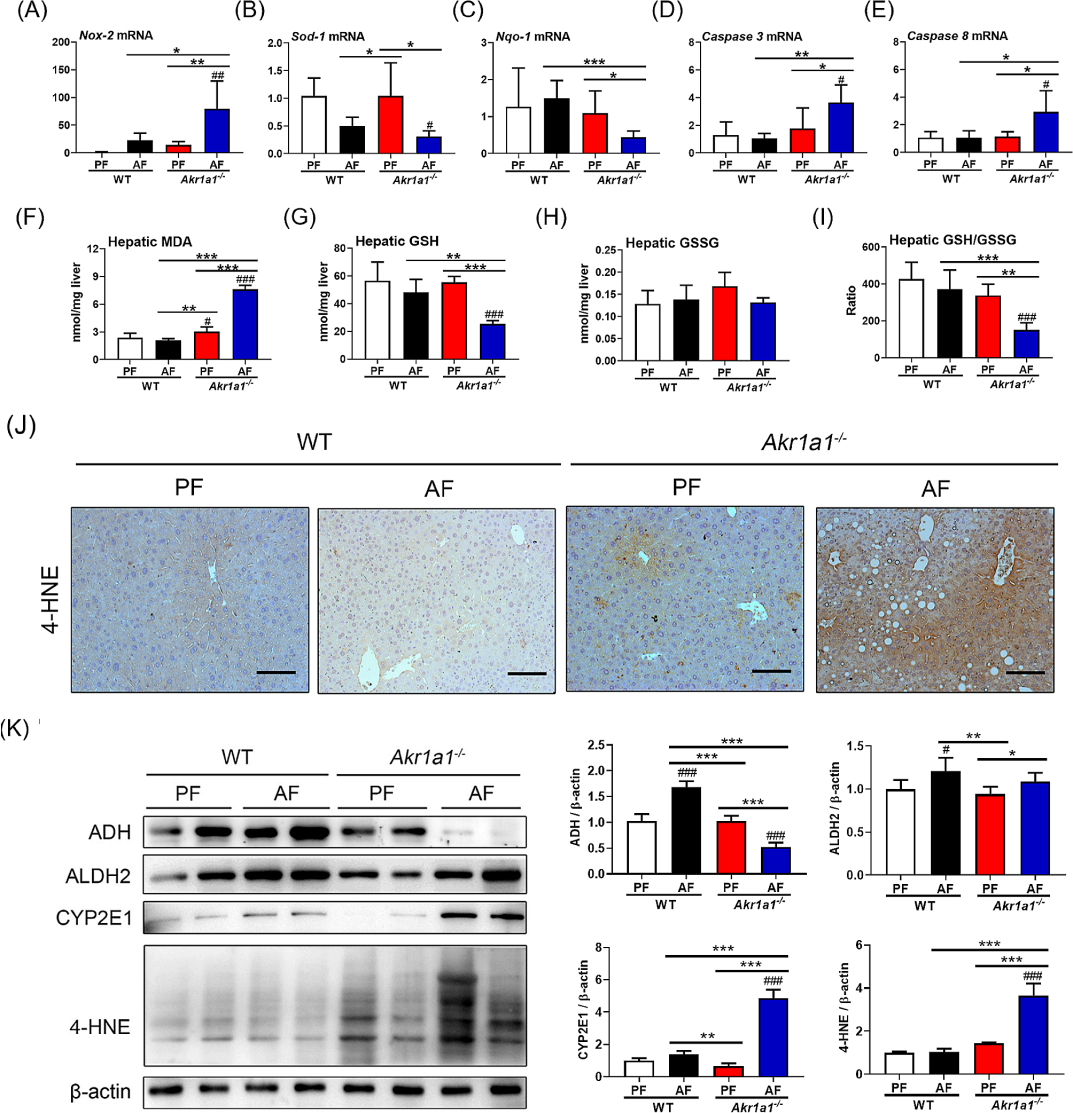
Loss of AKR1A1 aggravates alcohol-induced hepatocyte oxidative damage. The liver tissues were subjected to qRT‒PCR examination of the expression of *Nox-2* (**A**), *Sod-1* (**B**), *Nqo-1*(**C**), *Caspase 3* (**D**), and *Caspase 8* (**E**) and biochemical assays of MDA (**F**), GSH (**G**), and GSSG (**H**) levels. The calculated hepatic GSSG/GSH ratio is shown in Panel (**I**). The liver tissue sections were also subjected to IHC staining for the detection of 4-HNE (**J**). In addition, western blot analysis was performed to examine the hepatocellular ADH, ALDH2, CYP2E1, and 4-HNE levels (**K**). Data are presented as the means ± SD (*n* = 6). Statistical differences were marked as follows: ^#^*p* < 0.05, ^##^*p* < 0.01, ^###^*p* < 0.001 (compared with the PF-treated WT group); *p < 0.05, **p < 0.01, ***p < 0.001 (compared between the other groups)

We further assessed the effects of AKR1A1 deficiency on the expression of oxidative stress-related pro-oxidant (*Nox-2*) and antioxidant genes (*Sod-1*, *Nqo-1*) and apoptosis-related caspase genes (*Caspase 3*, *Caspase 8)* at the transcriptional level (Fig. 3A–E). As shown, AF treatment generally caused higher *Nox-2* expression than PF treatment, but this effect was particularly pronounced in *Akr1a1*^*−/−*^ mice compared to WT mice (Fig. 3A). The expression of *Sod-1* and *Nqo-1* was opposite to that of *Nox-2*; AF treatment significantly inhibited the transcription of *Sod-1* and *Nqo-1* in the livers of *Akr1a1*^*−/−*^ mice compared to WT mice (Fig. 3B, C). Furthermore, mice in the AF-treated *Akr1a1*^*−/−*^ group exhibited notable *caspase 3* and *caspase 8* expression compared to those mice in the other groups (Fig. 3D, E).

Moreover, the hepatic contents of MDA, a byproduct resulting from lipid peroxidation, were noticeably increased in the AF-treated *Akr1a1*^*−/−*^ group but remained low in the other groups (Fig. 3F). However, the hepatic levels of GSH, an endogenous thiol antioxidant, were remarkably depleted in the AF-*Akr1a1*^*−/−*^ group. The GSSG levels were similar among all groups, and thus, the GSSG/GSH ratio was markedly higher in the AF-*Akr1a1*^*−/−*^ group than in the other groups (Fig. 3G–I). Furthermore, intense signals of 4-hydroxynonenal (4-HNE), a reliable biomarker of lipid peroxidation in liver diseases [29], were detected in the liver tissue sections of AF-treated *Akr1a1*^*−/−*^ mice, whereas relatively weak signals were observed in the liver tissue of mice in the other groups (Fig. 3J). Western blot data further confirmed the high expression of 4-HNE in AF-treated *Akr1a1*^*−/−*^ mice (Fig. 3K). In addition, the western blot data demonstrated that the expression of ADH, which plays a role in the conversion of alcohol to acetaldehyde, was significantly inhibited in AF-treated *Akr1a1*^*−/−*^ mice, but CYP2E1, which works alongside ADH in alcohol metabolism, was expressed at a relatively higher level in the AF-treated *Akr1a1*^*−/−*^ group than in the other groups (Fig. 3K). Meanwhile, ALDH2, which plays an important role downstream of ADH and CYP2E1 by converting the resultant acetaldehydes to acetate in the mitochondria, was slightly increased in both AF-treated WT and *Akr1a1*^−/−^ mice than those of their PF-treated counterparts, with a similar trend (Fig. 3K). Taken together, the results demonstrated that the loss of AKR1A1 function increases alcohol-induced oxidative stress in the livers of *Akr1a1*^*−/−*^ mice.

### Loss of AKR1A1 function promotes alcohol-induced liver steatosis

**Fig. 4:**
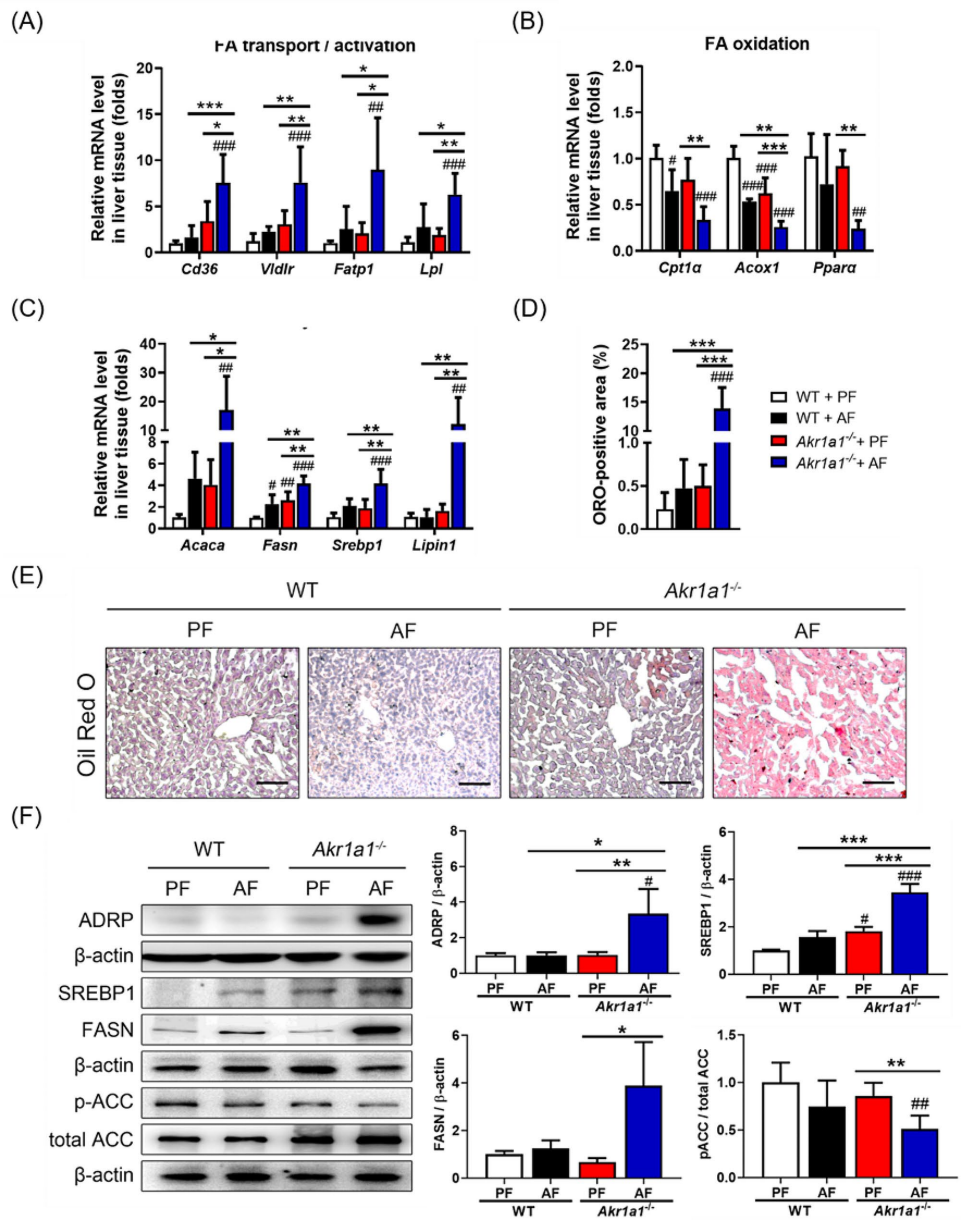
Loss of AKR1A1 aggravates alcohol-induced liver steatosis. The mRNA levels of the related marker genes involved in fatty acid (FA) transport and activation (*Cd36*, *Vldlr*, *Fatp1*, and *Lpl*) (**A**), FA oxidation (*Cpt1α*, *Acox1*, and *Pparα*) (**B**), and FA synthesis (*Acaca*, *Fasn*, *Srebp1*, and *Lipin1*) (**C**) were examined by qRT‒PCR to evaluate the effects of AKR1A1 loss on alcohol-induced liver steatosis. Values were normalized to the β-actin gene and expressed in relation to the PF-treated WT mice. The frozen liver sections were subjected to Oil Red O (ORO) staining (**E**), and the relative fat accumulation was compared by the quantification of ORO-positive stained areas (**D**). Western blot analysis of ADRP, SREBP1, FASN, and pACC/ACC expression in the liver is shown in Panel (**F**), in which the relative protein levels were quantified by β-actin normalization. Data are presented as the means ± SD (*n* ≥ 3). Statistical differences are marked as follows: ^#^*p* < 0.05, ^##^*p* < 0.01, ^###^*p* < 0.001 (compared with the PF-treated WT group); *p < 0.05, **p < 0.01, ***p < 0.001 (compared between the other groups)

To evaluate alcohol-induced fat accumulation in the liver, the expression of the related genes involved in fatty acid (FA) transport and activation (*Cd36*, *Vldlr*, *Fatp1*, and *Lpl*), FA oxidation (*Cpt1α*, *Acox1*, and *Pparα*), and FA synthesis (*Acaca*, *Fasn*, *Srebp1*, and *Lipin1*) was inspected at the transcriptional level (Fig. 4A–C). As shown, all of the examined genes involved in FA transport and activation (Fig. 4A) and FA synthesis (Fig. 4C) were markedly upregulated, while those contributing to FA oxidation (Fig. 4B) were significantly downregulated in the livers of AF-treated *Akr1a1*^*−/−*^ mice compared to mice in the other groups. ORO staining further demonstrated extensive fat accumulation in the liver tissue sections of AF-treated *Akr1a1*^*−/−*^ mice (Fig. 4D, E). Furthermore, as determined by western blotting, several proteins associated with FA synthesis, such as ADRP, SREBP1, and FASN, were overexpressed in the livers of AF-treated *Akr1a1*^*−/−*^ mice, while ACC phosphorylation (ACC inactivation) was significantly inhibited (Fig. 4F). These data demonstrated that loss of AKR1A1 function promotes alcohol-induced liver steatosis in *Akr1a1*^*−/−*^ mice.

### Loss of AKR1A1 function exacerbates alcohol-induced liver fibrosis

**Fig. 5:**
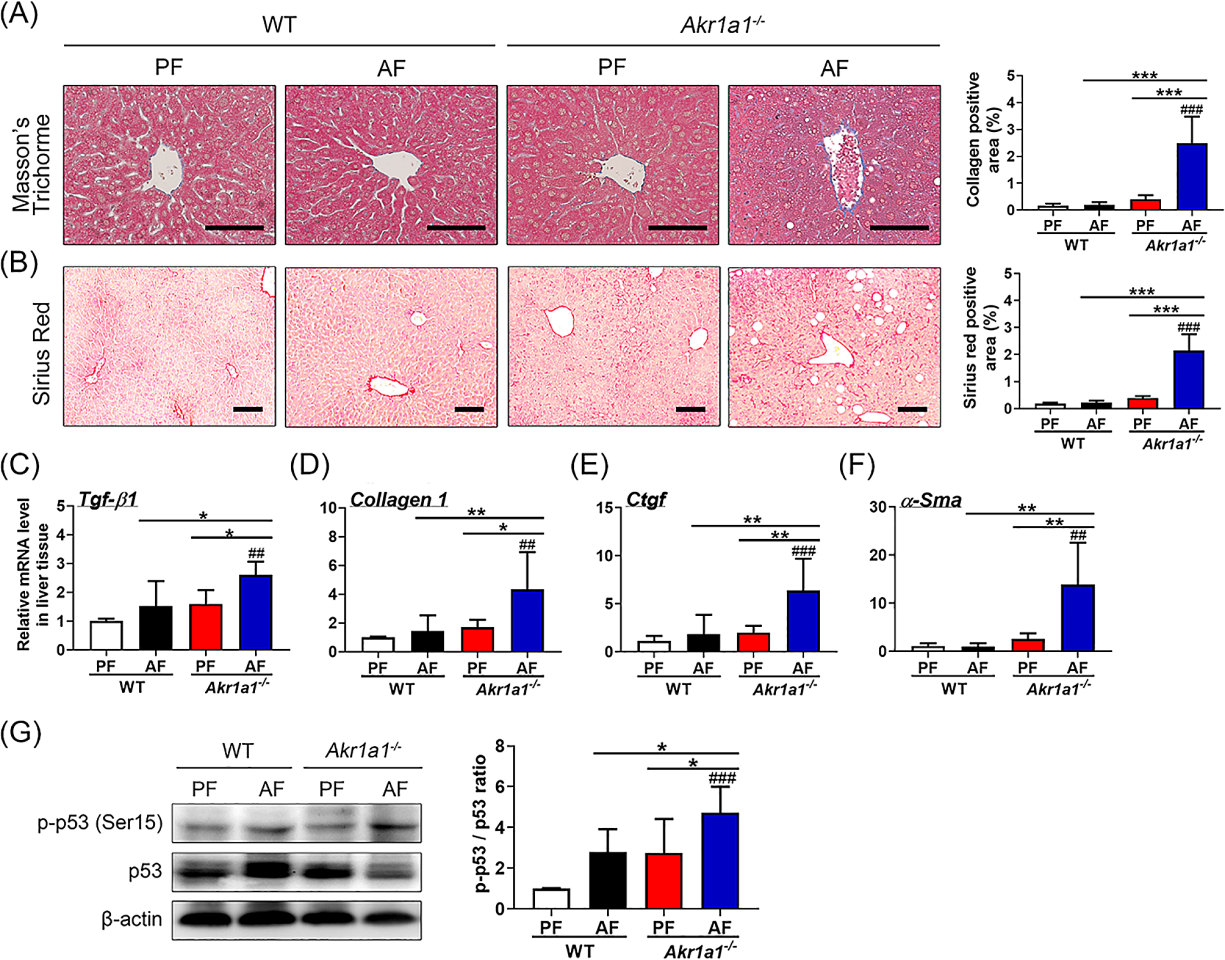
Loss of AKR1A1 aggravates alcohol-induced liver fibrosis. After the treatments, Masson’s trichrome (**A**) and Sirius red (**B**) staining were performed in the liver tissue sections to evaluate the effects of AKR1A1 loss on alcohol-induced liver fibrosis. Scale bar = 100 μm. The expression of fibrosis-related marker genes, including *Tgf-β1* (**C**), *Col1a1* (**D**), *Ctgf* (**E**), and *α-sma* (**F**), was examined by qRT–PCR. The relative mRNA levels were quantified by *β-actin* normalization and expressed in relation to the PF-treated WT mice. Furthermore, western blot analysis of the phosphorylated p53 (p-p53) and the total p53 in the liver tissues is shown in Panel (**G**). The relative p53 activation was quantified by determining the intensity ratio between p-p53 and p53 and subsequently comparing with the PF-treated WT mice. Data are presented as the means ± SD (*n* ≥ 5). Statistical differences were marked as follows: ^#^*p* < 0.05, ^##^*p* < 0.01, ^###^*p* < 0.001 (compared with the PF-treated WT group); *p < 0.05, **p < 0.01, ***p < 0.001 (compared between the other groups)

Next, liver fibrosis was examined by Masson’s trichrome and Sirius red staining, which showed that a particularly high amount of well-defined collagen fibers was found in the liver sections of AF-treated *Akr1a1*^*−/−*^ mice (Fig. 5A, B). In addition, fibrosis-related marker genes, including *Tgf-β1*, *Collagen I*, *Ctgf*, and *α-Sma*, were transcribed at a noticeably high level in the livers of AF-treated *Akr1a1*^*−/−*^ mice compared to mice in the other groups (Fig. 5C–F). Interestingly, the phosphorylation of p53 was increased in the liver tissues of AF-treated *Akr1a1*^*−/−*^ mice compared to those in the AF-treated WT mice or PF-treated *Akr1a1*^*−/−*^ mice (Fig. 5G). These data demonstrate that loss of AKR1A1 function exacerbates alcohol-induced liver fibrosis in *Akr1a1*^*−/−*^ mice and suggestes the involvement of p53-mediated signaling.

### *Akr1a1* knockdown aggravates alcohol-induced steatosis, inflammation, and fibrosis in AML12 hepatocytes

**Fig. 6:**
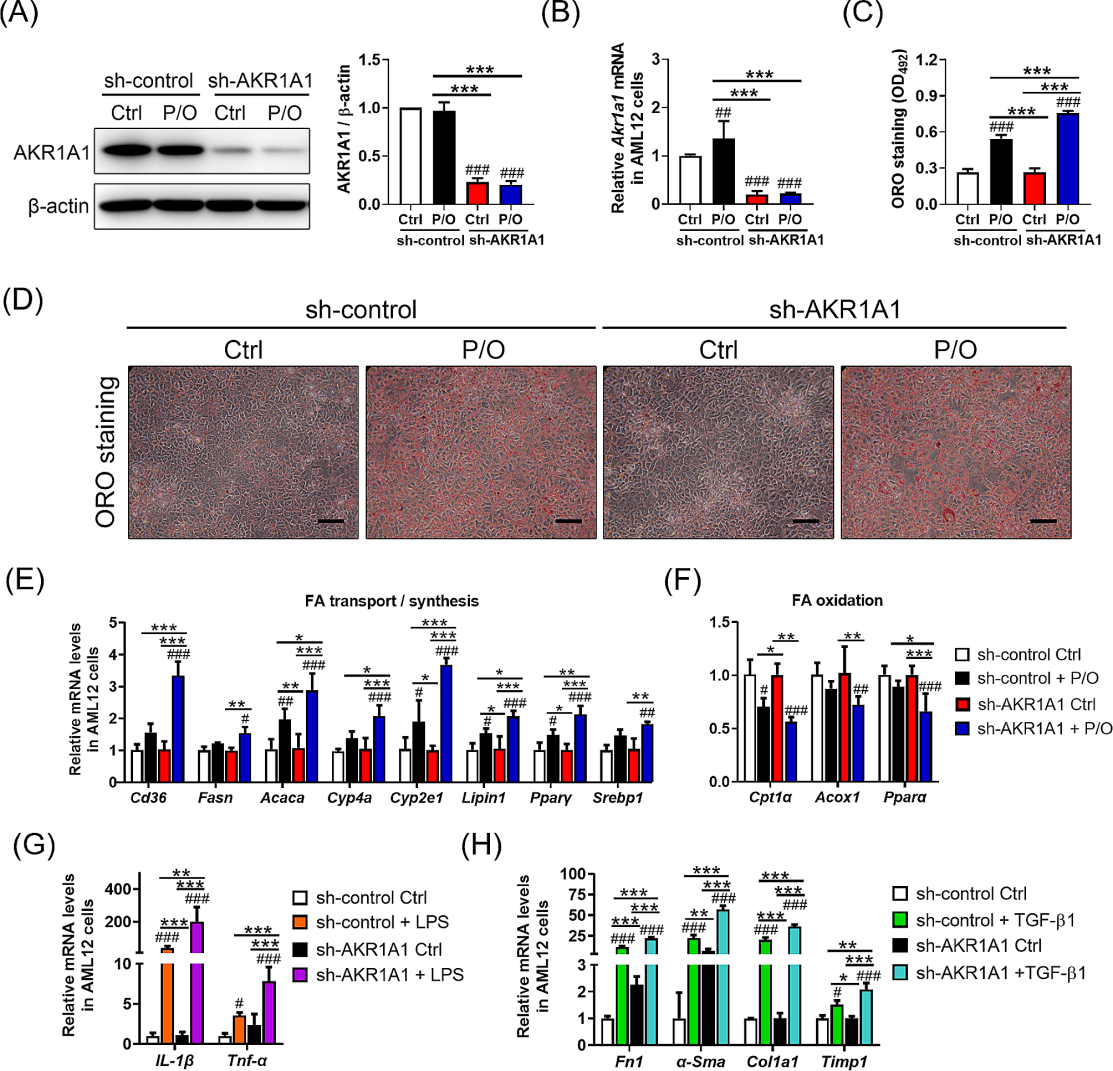
AKR1A1 knockdown in AML12 hepatocytes exacerbates lipid accumulation, inflammation, and fibrosis. AKR1A1 knockdown was conducted in AML12 hepatocytes by infecting cells with either a control (sh-control) or an AKR1A1-specific (sh-AKR1A1) shRNA lentiviral clone for 48 h. The shRNA-treated AML12 hepatocytes were then subjected to stimulation with a palmitic acid and oleic acid mixture (P/O) to induce steatosis in the absence or presence of 200 mM ethanol for 72 h. Afterward, western blotting and qRT‒PCR were performed to characterize AKR1A1 expression at both the protein (**A**) and mRNA (**B**) levels. ORO staining was performed to examine lipid accumulation in AML12 hepatocytes (**D**). The extent of lipid accumulation was quantified by extracting the bound ORO dye and detecting the absorbance at 450 nm (**C**). Furthermore, the related marker genes involved in FA transport and synthesis (*Cd36*, *Fasn*, *Acaca*, *Cyp4a*, *Cyp2e1*, *Lipin1*, *Ppar-γ*, and *Srebp1*) (**E**) and FA oxidation (*Cpt1α*, *Acox1*, and *Pparα*) (**F**) were examined at the mRNA level. In addition, shRNA-treated AML12 hepatocytes were stimulated with 1 µg/ml of LPS to induce inflammation for 24 h and stimulated with 5 ng/ml TGF-β1 to induce fibrosis for 48 h. After the treatments, qRT‒PCT was performed to characterize the mRNA levels of inflammation-related (*Il-1b*, *Tnf-α*) (**G**) and fibrosis-related (*Fn1*, *α-Sma*, *Col1a1*, and *Timp-1*) (**H**) marker genes. All values were normalized to the *β-actin* gene and expressed in relation to the sh-control group. Data are presented as the means ± SD (*n* ≥ 3). Statistical differences were marked as follows: ^#^*p* < 0.05, ^##^*p* < 0.01, ^###^*p* < 0.001 (compared with the sh-control Ctrl group); *p < 0.05, **p < 0.01, ***p < 0.001 (compared between the other groups)

The importance of AKR1A1 function in alcohol metabolism and ALD was further verified in mouse AML12 hepatocytes. Stimulation by the mixture of palmitic acid and oleic acid (P/O), LPS, and TGF-β1 was used to mimic alcohol-induced steatosis, inflammation, and fibrosis, and *Akr1a1* expression was knocked down by infection with a shRNA lentivirus targeting the *Akr1a1* gene (Fig. 6). The efficiency of *Akr1a1* knockdown in the present study was demonstrated to achieve approximately 80% inhibition of protein (Fig. 6A) and mRNA expression (Fig. 6B). Following *Akr1a1* knockdown by a control shRNA (sh-control), ORO staining revealed that red-stained lipid accumulation was increased in P/O-treated AML12 cells compared to control cells; however, when *Akr1a1* was knocked down by an *Akr1a1-*specific shRNA (sh-AKR1A1), the P/O-treated effect on lipid accumulation reached its highest level (Fig. 6C, D). Consistently, P/O treatment increased the mRNA levels of *Cd36*, *Fasn*, *Acaca*, *Cyp4a*, *Cyp2e1*, *Lipin1*, *Ppar-γ*, and *Srebp1* (marker genes involved in FA transport and synthesis) in AML12 hepatocytes, whereas the related mRNA levels were further elevated upon *Akr1a1* knockdown (Fig. 6E). Conversely, P/O treatment reduced the mRNA levels of *Cpt1α*, *Acox1*, and *Pparα* (marker genes involved in FA oxidation) in AML12 hepatocytes, whereas the related mRNA expression levels were further inhibited when *Akr1a1* was knocked down (Fig. 6F). Likewise, *Akr1a1* knockdown enhanced LPS-induced inflammatory responses in AML12 hepatocytes, as demonstrated by significantly increased *Il-1β* and *Tnf-α* mRNA levels (Fig. 6G). *Akr1a1* knockdown also enhanced TGF-β1-induced fibrotic responses in AML12 hepatocytes by increasing the mRNA levels of *Fn1*, *α-Sma*, *Col1a1*, and *Timp-1*, which are marker genes for the production of extracellular matrix (Fig. 6H). These data provide in vitro evidence showing that *Akr1a1* knockdown aggravates alcohol-induced steatosis, inflammation, and fibrosis.

## Discussion

ALD is one of the most common causes of cirrhosis globally due to heavy alcohol use, and related gene or protein markers for the purpose of diagnosis and therapeutic intervention must be extensively investigated. AKR1A1, or aldehyde reductase, is known to display enzymatic activities toward endogenous aldehydes and acts as a detoxifying enzyme in the liver. However, whether it contributes to alcohol metabolism and its association with ALD remain unclear. Data mining from earlier studies indicated the downregulation of AKR1A1 in clinical patients with ALD (Supplementary Table S3, Table S4, and Fig. S1) [24–26], and WT mice in the present chronic alcohol feeding model exhibited overexpression of AKR1A1 in the liver without observable liver damage, suggesting the protective role of AKR1A1 against ALD progression (Fig. 1B‒D). This presumption gained further support from the findings that *Akr1a1*^*−/−*^ mice presented a higher mortality rate than WT mice when they were exposed to long-term alcohol feeding (Fig. 2A). Additionally, serum analysis of the liver function indexes (AST, ALT) indicated liver dysfunction in alcohol-fed *Akr1a1*^*−/−*^ mice (Fig. 2C, D), and the results of histopathological staining further demonstrated that the loss of AKR1A1 function led to aggravated alcohol feeding-induced steatosis, fat accumulation, and fibrosis in their livers (Figs. 2G, 4D and E and 5A and B). In vitro, P/O-treated AML12 hepatocytes showed a similar increase in alcohol-induced fat accumulation when the expression of AKR1A1 was inhibited through the RNAi technique (Fig. 6C, D). Overall, alcohol-fed *Akr1a1*^*−/−*^ mice showed increased expression of genes involved in fatty acid transport, activation, and synthesis and decreased expression of genes involved in fatty acid β-oxidation (Fig. 4A‒C, F). Similar gene expression profiles were also observed in vitro when intracellular AKR1A1 levels were downregulated (Fig. 6E, F). The regulatory mechanism of *Akr1a1* expression in response to alcohol consumption remains currently unknown. We can anticipate that alcohol consumption induces AKR1A1 protein expression and other protective enzymes in the liver tissue in order to reduce the alcohol-induced increase in oxidative stress and inflammation. If these adverse cellular reactions cannot be released, oxidative damage is supposed to occur. Oxidative damage has been linked to single nucleotide polymorphisms (SNPs) of the *Akr1a1* gene in a number of pathological conditions [30], suggesting *Akr1a1* is a “hot spot” of genetic mutation in response to environmental or physiological stimuli. From a healthy state to progressing into ALD, it is usually a dose- and time-dependent process that also depends on an individual’s sensitivity to alcohol. Once the alcohol-induced oxidative damage overload occurs, it may accumulate varied mutations in the promoter or coding regions of the *Akr1a1* gene, thus decreasing the expression and activities of AKR1A1. This surmise coupling with the observation of hypermethylation in the *Akr1a1* promoter of cirrhotic patients may explain why *Akr1a1* downregulation is usually observed in ALD patients (Supplementary Table S3, Table S4, and Fig. S1). So far, SMAR1 is the only regulator for *Akr1a1* expression. Earlier study reported that SMAR1 acts as a tumor suppressor that involved in cell cycle regulation and inhibits AKR1A4 (the synonym of AKR1A1) via a direct interaction [31]. However, whether SMAR1 or other regulators are involved in alcohol-induced *Akr1a1* expression requires further studies.

Mechanistically, the loss of AKR1A1 function resulted in an increase in oxidative stress in the liver by affecting the balance of cellular pro- and antioxidants (Fig. 3A‒C, F‒I). It also aggravated alcohol-induced liver inflammation by increasing the release of TNF-α and IL-1β (Fig. 2H, I), two important proinflammatory cytokines, as well as promoting the apoptosis of hepatocytes (Fig. 3D, E). It is worth noting that loss of AKR1A1 function increased intracellular 4-HNE levels in the livers of *Akr1a1*^*−/−*^ mice receiving long-term alcohol feeding (Fig. 3K). 4-HNE is a primary α,β-unsaturated hydroxyalkenal that is generated in higher quantities due to the increase in lipid peroxidation and is the most toxic form of aldehyde that can accelerate cellular damage caused by unrestricted oxidative stress or the formation of nucleic acid and protein adducts through Michael addition or Schiff base formation [32, 33]. Higher intracellular content of 4-HNE (around several tens of µM) has been shown to trigger reactive oxygen species (ROS) generation, deplete the antioxidant capacity of the cells, and activate diverse pathways, such as inflammation, apoptosis, and necrosis, with the eventual outcome of cell death [32]. Thus, it is linked to the pathology of neurodegenerative diseases (such as Alzheimer’s, Huntington’s, and Parkinson’s diseases) [32, 34], cancers [35], myocardial diseases [36], pulmonary diseases [37], nonalcoholic and alcoholic fatty liver diseases [38, 39], etc. Several mechanisms have been reported to explain the possible actions of 4-HNE on liver damage. One mechanism was identified in the study of Dou et al. [38], in which they demonstrated that long-term alcohol feeding led to the costimulation of hepatic 4-HNE and TNF-α content in male C57BL/6 mice and the elevation of 4-HNE sensitized primary hepatocytes to TNF-α cytotoxicity through the inhibition of NF-κB activation. The second mechanism was identified in the study of Doorn et al. [39], which demonstrated that 4-HNE may act as a potent inhibitor of ALDH2 by forming adducts with it. The last mechanism was identified by Shearn et al. [40] in a study that suggested that 4-HNE-mediated PTEN inhibition and Akt2 activation is a novel mechanism of lipid accumulation in response to increased aldehyde production during chronic alcohol feeding. Whether these possible mechanisms work individually or in combination in the present animal model requires further investigation. Direct evidence that AKR1A1 is involved in the clearance of 4-HNE or other endogenous aldehydes is limited. An in vitro experiment indicated that recombinant human AKR1A1 protein displayed a specific activity of ~ 80 nmol/min/mg for the reduction of 4-HNE [41], and Liu et al. reported that the mRNA level of AKR1A1 was upregulated upon exposure to 4-HNE in porcine enterocytes [42]. However, these two studies suggested a major role for AKR1C1 instead of AKR1A1 in the clearance of 4-HNE. In our case, alcohol feeding increased AKR1A1 expression in the WT mice to maintain 4-HNE at a low level compared to the pair-fed WT mice. However, the alcohol-induced 4-HNE accumulation cannot be relieved in the *Akr1a1*^*−/−*^ mice due to the complete loss of AKR1A1, even though compensatory effects from the other enzymes of the AKR superfamily might exist (Figs. 2 and 3).

Furthermore, we also found that p53 phosphorylation was significantly increased in alcohol-fed *Akr1a1*^*−/−*^ mice, suggesting that the loss of AKR1A1 enhanced alcohol-induced p53 activation (Fig. 5G). p53 has been characterized as a tumor suppressor and has recently been recognized as a central metabolic regulator of diverse physiological and pathological processes. The activation of p53-mediated pathways has been reported in different pathological settings, many of which directed the cell fate toward apoptosis [33]. Pani et al. reported that no apoptosis was induced by ethanol in p53 knockout (p53^−/−^) mice, suggesting the role of p53 in mediating the harmful effects of ethanol on hepatocytes [43]. 4-HNE-induced DNA damage and oxidative stress were shown to trigger the intrinsic apoptotic pathway through p53 activation [33]. An earlier rat model of ALD demonstrated that p53 activation not only enhanced hepatocellular apoptosis but also promoted insulin resistance in the liver, contributing to the metabolic abnormalities associated with ALD [44]. Furthermore, we linked p53 activation to the upregulation of fibrosis-related marker genes encoding TGF-β1, collagen I, CTGF, and α-SMA in alcohol-fed *Akr1a1*^*−/−*^ mice, which was supported by a previous study showing that p53 activation upregulates hepatic CTGF expression, leading to liver fibrosis [45]. Moreover, high expression levels of hepatic p53 may induce ROS production, cell apoptosis, or necrosis, leading to tissue inflammation, which can contribute to subsequent liver steatosis and cirrhosis [45, 46]. In contrast, ablation of p53 has been shown to reduce liver cell apoptosis, oxidative stress, and lipid metabolism in chronic alcohol- (43,44) or nonalcohol-induced steatohepatitis mouse models [47, 48].

In addition, we found that alcohol feeding increased ADH, ALDH2, and CYP2E1 in WT mice, while the same treatment led to the inhibition of ADH and a much higher CYP2E1 in *Akr1a1*^*−/−*^ mice (Fig. 3K). Alcohol feeding also slightly increased ALDH2 in *Akr1a1*^*−/−*^ mice, but the effect was similar to that of WT mice (Fig. 3K), suggesting that *Akr1a1* knockout caused little or no influence on ALDH2 expression. It is interesting to find the remarkable inhibition of ADH in *Akr1a1*^*−/−*^ mice after the alcohol treatment. The cause of ADH inhibition in alcohol-treated *Akr1a1*^*−/−*^ mice is not understood yet. ADH is mainly expressed in the cytosol. Earlier experiments suggested that excess 4-HNE accelerates the ubiquitination and proteasomal degradation of ADH modified with 4-HNE [49]. Based on this, we speculated that the inhibition of ADH in alcohol-treated *Akr1a1*^*−/−*^ mice might be due to the lack of AKR1A1 activity leading to 4-HNE accumulation in the cytosol and thus enabling excess 4-HNE to form a protein adduct with ADH, which is mainly expressed in the cytosol. However, this situation may not happen in WT mice because ethanol will be efficiently metabolized, and thus 4-HNE will not accumulate. Due to the fact that ALDH2 and CYP2E1 are mainly mitochondrial and microsomal proteins, respectively, the compartment barriers may protect them from reacting with 4-HNE. Both ADH and CYP2E1 in the hepatocyte catalyze the transformation of ethanol into acetaldehyde, a highly reactive metabolite that can form stable and covalent adducts with intracellular proteins or other macromolecules [50]. However, CYP2E1 catalyzes not only the production of acetaldehyde but also the production of highly reactive oxygen radicals, contributing to enhanced oxidative stress and lipid peroxidation, which in turn promote the generation of reactive metabolites similar to acetaldehyde, especially MDA and 4-HNE [50]. In our case, the effect of CYP2E1 upregulation overwhelmed the effect of ADH reduction in *Akr1a1*^*−/−*^ mice. This suggests that *Akr1a1*^*−/−*^ mice are prone to accumulate higher oxidative stress in the liver after alcohol feeding and shows the indirect action of AKR1A1 in regulating alcohol metabolism. In mice, AKR1A1 also contributes to the biosynthesis of vitamin C, so the loss of AKR1A1 is equivalent to a reduction in the antioxidant capacity of vitamin C. This may partly explain why *Akr1a1*^*−/−*^ mice are prone to liver injuries after alcohol feeding. In this regard, several studies have demonstrated that replenishing vitamin C via its antioxidative capability is effective in preventing drug-induced liver lesions in *Akr1a1*^*−/−*^ mice [13–17].

**Fig. 7:**
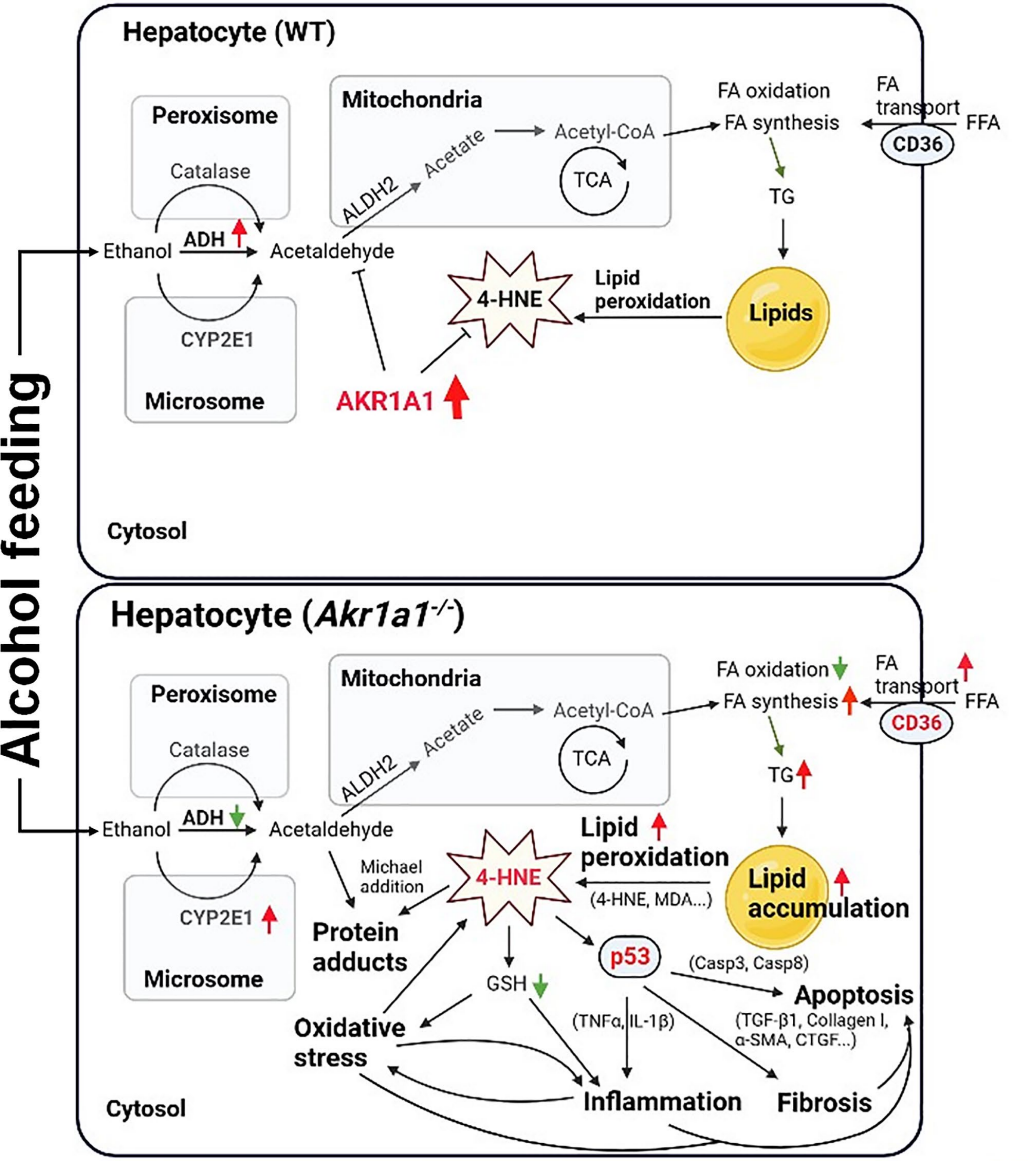
The proposed mechanism shows that AKR1A1 functions in a liver-protective manner after extended alcohol drinking by lowering the accumulation of acetaldehyde and 4-HNE in hepatocytes

In summary, as shown in Fig. 7, we propose that AKR1A1 functions in a liver-protective manner after extended alcohol consumption, predominantly by decreasing the accumulation of 4-HNE due to increased lipid peroxidation. This action not only alleviates the oxidative stress brought about by alcohol metabolism but also reduces the hepatocellular damage caused by acetaldehyde and 4-HNE adducts and weakens 4-HNE-mediated p53 activation in subsequent hepatocellular apoptosis, fibrosis, and inflammation. Moreover, we found that alcohol feeding raised hepatic ADH levels in the presence of AKR1A1 but inhibited ADH expression with an elevation in CYP2E1 levels in the absence of AKR1A1.

## Conclusions

Although previous studies found that AKR1A1 levels are decreased in the livers of patients with alcoholic liver disease, no research has further explored its correlation with the development of the disease. In this study, we address the protective roles of AKR1A1 in the hepatic metabolism of alcohol and demonstrate the related liver injuries caused by chronic alcohol feeding in the context of *Akr1a1* knockout. The present data suggest that AKR1A1 mainly plays a role in clearing the main intermediates of alcohol metabolism, acetaldehyde and 4-HNE. This not only leads to a reduction in intracellular oxidative stress caused by these metabolites but also reduces their further reactions to form protein adducts with other intracellular macromolecules and 4-HNE-mediated p53 activation, which may further amplify the hepatocellular damage.

### Electronic supplementary material

Below is the link to the electronic supplementary material.

Supplementary Material 1

## Data Availability

Data will be available on request.

## Abbreviations

4-HNE: 4-hydroxynonenal
ADH: Alcohol dehydrogenase
ADRP: Adipose differentiation-related protein
AF: Alcohol-fed
AKR1A1: Aldo-keto reductase family 1 member A1
ALD: Alcoholic-associated liver disease
ALT: Alanine aminotransferase
AST: Aspartate aminotransferase
Casp3: Caspase 3
Casp8: Caspase 8
Col1a1: Collagen type I alpha 1
Ctgf: Connective tissue growth factor
CYP2E1: Cytochrome p450 family 2 subfamily E member 1
FASN: Fatty acid synthase
GSH: Reduced glutathione
GSSG: Oxidized glutathione
H&E: Hematoxylin and eosin
IHC: Immunohistochemistry
IL-1β: Interleukin-1β
MDA: Malondialdehyde
Nox-2: NADPH oxidase 2
Nqo-1: NAD(P)H quinone dehydrogenase 1
ORO: Oil red O
pACC: Phospho-acetyl-CoA carboxylase
PF: Pair-fed
qRT-PCR: Quantitative real-time reverse transcription polymerase chain reaction
Sod-1: Superoxide dismutase type 1
SREBP1: Sterol regulatory element-binding transcription factor 1
Tgf-β1: Transforming growth factor beta 1
TNF-α: Tumor necrosis factor-α
WT: Wild type

## Data availability

All data generated or analyzed during the current study are available from the corresponding author upon reasonable request.
