# Supplementary material for: Aldo-keto reductase family 1 member A1 (AKR1A1) exerts a protective function in alcohol-associated liver disease by reducing 4-HNE accumulation and p53 activation
Source: Cell Biosci. 2024 Feb 3;14:18. doi: 10.1186/s13578-024-01200-0 (PMC10837880; doi:10.1186/s13578-024-01200-0)
Supplement: Supplementary file 1 — Supplementary Material 1 [file 13578_2024_1200_MOESM1_ESM.pdf]

Supplementary Materials

Supplementary Table S1. Primers used for qRT–PCR

Target	Sense	Antisense
IL-1β	5'-GCTCATCTGGGATCCTCTCC-3'	5'-CCTGCCTGAAGCTCTTGTTG-3'
TNF-α	5'-CCCTCACACTCAGATCATCTTCT-3,	5'-GCTACGACGTGGGCTACAG-3'
NOX-2	5'-AGTGCGTGTTGCTCGACAA3	5'-GCGGTGTGCAGTGCTATCAT-3'
SOD-1	5'-TATGGGGACAATACACAAGGCT-3'	5'-CGGGCCACCATGTTTCTTAGA-3'
NQO-1	5'-AGAGAGTGCTCGTAGCAGGAT-3'	5'-GTGGTGATAGAAAGCAAGGTCTT-3'
Casp3	5'-CTGACTGGAAAGCCGAAACTC-3'	5'-CGACCCGTCCTTTGAATTTCT-3'
Casp8	5'-CAACTTCCTAGACTGCAACCG-3'	5'-TCCAACTCGCTCACTTCTTCT-3'
CD36	5'-ATGGGCTGTGATCGGAACTG-3'	5'-TTTGCCACGTCATCTGGGTTT-3'
VLDLR	5'-TGATTGCGAAGACGGTTCTGA-3'	5'-CCAGGACACGGGGATACACT-3'
FATP1	5'-CGCTTTCTGCGTATCGTCTG-3'	5'-GATGCACGGGATCGTGTCT-3'
LPL	5'-GGGAGTTTGGCTCCAGAGTTT-3'	5'-TGTGTCTTCAGGGGTCCTTAG-3'
CPT1α	5'-AGATCAATCGGACCCTAGACAC-3'	5'-CAGCGAGTAGCGCATAGTCA-3'
ACOX1	5'-TAACTTCCTCACTCGAAGCCA-3'	5'-AGTTCCATGACCCATCTCTGTG-3'
PPAR-α	5'-GCAGCTCGTACAGGTCATCA-3'	5'-CTCTTCATCCCCAAGCGTAG-3'
ACACA	5'-AATGAACGTGCAATCCGATTTG-3'	5'-ACTCCACATTTGCGTAATTGTTG-3'
FASN	5'-GGCTCTATGGATTACCCAAGC-3'	5'-CCAGTGTTCGTTCCTCGGA-3'
SREBP1	5'-GATCGCAGTCTGAGGAGGAG-3'	5'-GATAGCAGGATGCCAACAGC-3'
LIPIN1	5'-CATGCTTCGGAAAGTCCTTCA-3'	5'-GGTTATTCTTTGGCGTCAACCT-3'
CYP2E1	5'-GGACCTTTCCCAATTCCTTTCTT-3'	5'-TCTTGTGGTTCAGTAGCACCT-3'
CYP4A	5'-TTCCCTGATGGACGCTCTTTA-3'	5'-GCAAACCTGGAAGGGTCAAAC-3'
PPAR-γ	5'-TGTGGGGATAAAGCATCAGGC-3'	5'-CCGGCAGTTAAGATCACACCTAT-3'
TGF-β1	5'-CTTCAATACGTCAGACATTCGGG-3'	5'-GTAACGCCAGGAATTGTTGCTA-3'
COL1A1	5'-CTGACTGGAAGAGCGGAGAGTAC-3'	5'-ACAGACGGCTGAGTAGGGAACA-3'
CTGF	5'-ACCTGGAGGAAAACATTAAGAAGG-3'	5'-AGCCCTGTATGTCTTCACACTG-3'
α-SMA	5'-TCCTTCGTGACTACTGCCGAGC-3'	5'-AATGGTGATCACCTGCCCGTC-3'
FN1	5'-TTCAAGTGTGATCCCCATGAAG-3'	5'-CAGGTCTACGGCAGTTGTCA-3'
TIMP1	5'-GCAACTCGGACCTGGTCATAA-3'	5'-CGGCCCGTGATGAGAAACT-3'
AKR1A1	5'-CAACTGGAGTATTTGGACCTC-3'	5'-GACATCATCAATCTGCCGAC-3'
β-actin	5'-CGCCACCAGTTCGCCATGGA-3'	5'-TACAGCCCGGGGAGCATCGT-3'

Supplementary Table S2. Primary antibodies used for western blot analysis and IHC staining

Antibody	Dilution used for		Host	Type	Supplier
	WB	IHC			
**anti-AKR1A1**	1:2000	1:200	Rabbit	Polyclonal	Atlas Antibodies AB
**anti-IL-1β**	1:1000	-	Mouse	Monoclonal	Cell Signaling Technology
**anti-TNF-α**	1:2000	-	Mouse	Monoclonal	Proteintech
**anti-ADH**	1:5000	-	Mouse	Monoclonal	Santa Cruz
**anti-CYP2E1**	1:2000	-	Rabbit	Polyclonal	CUSABIO Technology
**anti-4-HNE**	1:2000	1:200	Rabbit	Polyclonal	Abeam
**anti-ADRP**	1:500	-	Rabbit	Polyclonal	Novus Biologicals
**anti-SREBP1**	1:500	-	Rabbit	Polyclonal	Santa Cruz
**anti-FASN**	1:500	-	Rabbit	Polyclonal	Santa Cruz
**anti-p-ACC (Ser79)**	1:1000	-	Rabbit	Monoclonal	Cell Signaling Technology
**anti-ACC**	1:2000	-	Rabbit	Polyclonal	GeneTex
**anti-p-p53 (Ser15)**	1:1000	-	Rabbit	Polyclonal	Cell Signaling Technology
**anti-p53**	1:1000	-	Mouse	Monoclonal	Cell Signaling Technology
**anti-β-actin**	1:10000	-	Mouse	Monoclonal	Novus Biologicals

Abbreviations: AKR1A1, aldo-keto reductase family 1 member A1; IL-1β, interleukin-1β; TNF-α, tumor necrosis factor-α; ADH, alcohol dehydrogenase; CYP2E1, cytochrome p450 family 2 subfamily E member 1; 4-HNE, 4-hydroxynonenal; ADRP, adipose differentiation-related protein; SREBP1, sterol regulatory element-binding transcription factor 1; FASN, fatty acid synthase; pACC, phospho-acetyl coA carboxylase.

Supplementary Table S3. Differential expression of the AKR1 family in alcoholic hepatitis (AH) patients (data mining from the NCBI GEO term GSE142530 [24])

Gene	p-value	Log2 fold change (AH/Normal)	Fold change (AH/Normal)
**AKR1A1**	**3.03E-01**	**– 0.31455**	**0.804**
AKR1B1	5.56E-02	0.44556	1.362
AKR1B10	6.66E-24	5.77366	54.707
AKR1B15	1.89E-14	4.53844	23.238
AKR1C1	4.25E-01	– 0.39091	0.763
AKR1C2	6.87E-01	0.21097	1.157
AKR1C3	1.47E-01	0.38514	1.306
AKR1C4	6.85E-01	– 0.22607	0.855
AKR1C8	4.39E-04	– 1.81057	0.285
AKR1D1	5.01E-03	– 1.81974	0.283
AKR1E2	1.42E-01	– 0.47585	0.719

Supplementary Table S4. Significantly differentially abundant proteins in the analysis of covariance (ANCOVA) in the liver proteome (data mining from the gut-and-liver axis–alcohol-related liver disease (GALA–ALD) cohort study [27])

Protein ID	Gene	Mean protein level at F0# (Log2 intensity)	Mean protein level at F1# (Log2 intensity)	Mean protein level at F2# (Log2 intensity)	Mean protein level at F3# (Log2 intensity)	Mean protein level at F4# (Log2 intensity)	Fold change (F2/F0)	Fold change (F3/F0)	Fold change (F4/F0)	ANCOVA significance combined	ANCOVA significance of fibrosis
P14550	AKR1A1	23.17	22.73	22.86	22.37	22.43	0.81	0.58	0.60	+	+

# denotes the fibrosis stages from F0 to F4 (F0: no fibrosis; ≥ F2: significant fibrosis; ≥ F3: advanced fibrosis).

Supplementary Table S5. Changes in the body, liver, and epididymal fat weights of WT and *Akr1a1*$^{-/-}$ mice with 8-week pair-fed (PF) or alcohol-fed (AF) treatments

Group	WT+ PF	WT + AF	*Akr1a1*$^{-/-}$ + PF	*Akr1a1*$^{-/-}$ + AF
Initial weight (g)	32.12 ± 1.39[a]	32.35 ± 1.79[a]	30.13 ± 3.05[a]	26.65 ± 2.95[b]
Final weight (g)	35.78 ± 1.82[a]	34.54 ± 1.18[a]	33.79 ± 2.91[a]	25.22 ± 2.60[b]
Liver weight (g)	1.55 ± 0.10[a]	1.57 ± 0.06[a]	1.42 ± 0.16[b]	1.81 ± 0.10[c]
Liver weight / final weight (%)	4.34 ± 0.29[a]	4.48 ± 0.15[a]	4.36 ± 0.20[a]	6.41 ± 0.80[b]
Epididymal fat weight (g)	0.52 ± 0.20[a]	0.27 ± 0.09[b]	0.40 ± 0.12[a]	0.20 ± 0.17[c]
Epididymal fat weight / final weight (%)	1.33 ± 0.43[a]	0.84 ± 0.23[b,c]	1.17 ± 0.32[a,b]	0.59 ± 0.52[d]

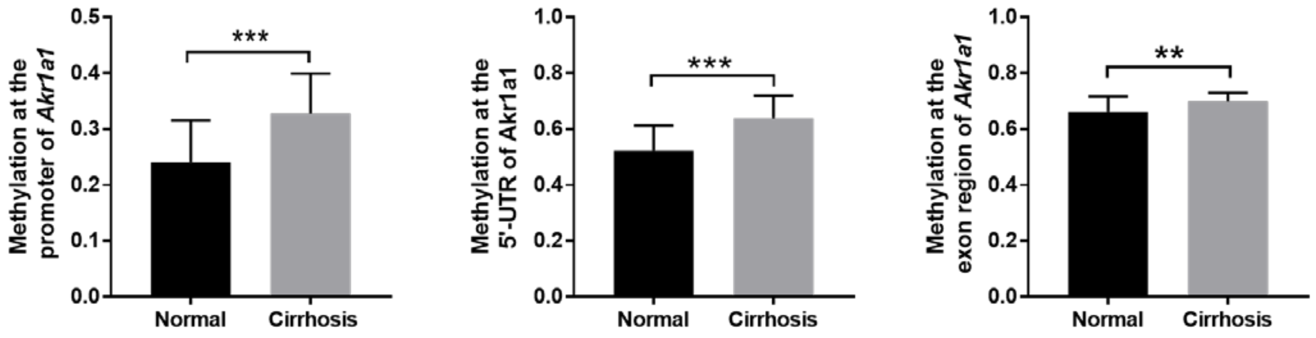

Supplementary Figure S1. Methylation in the promoter, 5'-UTR, and exon regions of the *Akr1al* gene. These data were mined from the study of Hlady et al. [25], in which the epigenetic signatures of alcoholism-induced cirrhosis (n = 21) were compared to those of normal livers (n = 34). The data are expressed as the mean ± SEM, **$p < 0.01$, and ***$p < 0.001$.
